# Endoscopic Versus Surgical Treatment for Ampullary Lesions: A Systematic Review With Meta-Analysis

**DOI:** 10.7759/cureus.65076

**Published:** 2024-07-22

**Authors:** Luiza Martins Baroni, Mateus Pereira Funari, Angelo So Taa Kum, Alexandre Moraes Bestetti, Luiza Bicudo de Oliveira, Matheus Ferreira de Carvalho, Tomazo Antonio Prince Franzini, Diogo Turiani Hourneaux de Moura, Wanderley Marques Bernardo, Eduardo Guimarães Hourneaux de Moura

**Affiliations:** 1 Gastroenterology, Hospital das Clínicas de São Paulo, São Paulo, BRA; 2 Gastrointestinal Endoscopy, Hospital Nove de Julho, São Paulo, BRA; 3 Gastrointestinal Endoscopy, Hospital das Clínicas da Faculdade de Medicina da Universidade de São Paulo, São Paulo, BRA; 4 Gastroenterology, Hospital das Clínicas da Faculdade de Medicina da Universidade de São Paulo, São Paulo, BRA; 5 Endoscopy, Hospital das Clínicas de São Paulo, São Paulo, BRA

**Keywords:** surgery, pancreaticoduodenectomy, endoscopy, duodenal neoplasms, ampulla of vater, ampullary adenoma

## Abstract

Ampullary lesions (ALs) can be treated through either an endoscopic approach (EA) or a surgical approach (SA). However, it is important to note that EAs carry a significant risk of incomplete resection, while opting for surgical interventions can result in substantial morbidity. We performed a systematic review and meta-analysis for R0 resection, recurrence, adverse events in general, major adverse events, mortality, and length of hospital stay between SAs and EAs. Electronic databases were searched from inception to 2023. We identified nine independent studies. The risk difference was -0.32 (95% CI: -0.50, -0.15; p <0.001) for R0, 0.12 (95% CI: 0.06, 0.19; p < 0.001) for recurrence, -0.22 (95% CI: -0.43, 0.00; p 0.05) for overall adverse events, -0.11 (95% CI: -0.32, 0.10; p = 0.31) for major complications, -0.01 (95% CI: -0.02, 0.01; p = 0.43) for mortality, and -14.69 (95% CI: -19.91, -9.47; p < 0.001) for length of hospital stay. As expected, our data suggest a higher complete resection rate and lower recurrence from surgical interventions, but this is associated with an elevated risk of adverse events and a longer hospital stay.

## Introduction and background

Neoplasia of the ampulla of Vater is an uncommon condition, with an annual incidence of fewer than 1 per 100,000 individuals [1-4]. Nevertheless, ampullary tumors are now being detected more frequently due to the enhanced precision of endoscopy and imaging methods. Given the malignant potential, complete removal of an adenoma and other neoplasms is imperative for curative therapy [5].

In the past, the only curative options for benign ampullary lesions (ALs) were either pancreatoduodenectomy (PD) [6] or surgical ampullectomy [7], both of which carry substantial morbidity and even mortality [8-10]. The introduction of advanced endoscopic ampullary resection has emerged as the preferred treatment for specific benign ampullary tumors. This preference is attributed to the treatment’s lower morbidity and considerable efficacy [11,12].

Tumor size can offer direction for therapy selection and serve as a predictor of endoscopic outcomes. Given conflicting findings in this regard among current studies, the management of ALs relies on local expertise [1]. The endoscopic approach (EA) is typically conducted for smaller lesions that show no signs of invasive carcinoma, exhibit clear margins, have a soft tissue texture, and are free from ulceration [3,13]. On the other hand, surgery is recommended when malignant findings are present on either endoscopic or pathology findings or when preoperative imaging indicates invasion of the biliary or the pancreatic duct. However, in some instances, due to the patient's clinical condition, individuals who would have been considered for surgery are instead offered an EA, primarily due to the lower morbidity [13].

Today, there is still only a limited number of studies, and they are retrospective cohorts comparing surgery versus endoscopy in the treatment of benign ALs. These studies exhibit varying inclusion criteria, outcomes, and surgical approaches (SAs). Therefore, the objective of this systematic review and meta-analysis was to compare the outcomes of the EA and the SA for benign ALs.

## Review

Methods

Protocol and Registration

This study was performed according to PRISMA guidelines (Preferred Reporting Items for Systematic Reviews and Meta-Analysis) [14] and registered in PROSPERO (International Prospective Register of Systematic Reviews) under the register CRD42018109713.

Study Identification and Selection

The systematic review included the MEDLINE, Embase, and LILACS databases. The search strategy was based on the MESH terms: (Adenoma OR Vater Ampulla OR Hepatopancreatic Ampulla OR Duodenal Papilla OR Bile Duct Neoplasms) AND (Endoscopic or endoscopy or endoscopies) AND (Pancreaticoduodenectomy OR Pancreaticoduodenectomies OR Duodenopancreatectomies OR Duodenopancreatectomy).

Data Collection Process

Only studies involving human subjects were considered for the analysis. Any retrospective or prospective study that compared EA versus PD for ALs and reported at least one of the specified outcomes was included. The primary outcome was the rate of complete resection (R0), determined by histology. Secondary outcomes included recurrence, overall adverse event rates, major adverse events, mortality, and length of hospital stay. Recurrence was defined as the appearance of a new lesion on endoscopy following initial negative follow-up endoscopy in EA and SA or the identification of local or distant recurrence in cross-sectional imaging in EA and SA. Major complications for both endoscopic and surgical interventions were defined by a Clavien-Dindo classification ≥III. According to this system, complications are divided into grades. Grade I comprises mild complications that do not require further treatment, other than simple care such as dressings or oral medication; grade II - complications that necessitate additional pharmacological treatment along with routine care, such as antibiotics, analgesics, antiemetics, and/or transfusions; grade III - severe complications that require surgical, radiological, or endoscopic intervention; these complications may include abscess drainage, surgical revisions, or other invasive procedures; grade IV - complications that threaten the patient's life and demand intensive care or substantial surgical interventions for correction; and grade V - patient death due to a procedural complication [15]. Only cases of procedure-related mortality were considered.

Statistical Analysis

The risk differences (RD) of the dichotomous outcomes were calculated using the Mantel-Haenszel test, and the mean difference (MD) was calculated using the inverse variance for continuous outcomes, with a 95% confidence interval (CI). Heterogeneity was reported by Chi-squared (Χ²) and I2. A random-effects model was used, as the studies showed high heterogeneity (I2 > 50). When heterogeneity was low, a fixed-effects model was employed. Continuous outcomes that initially presented median and range were converted to mean and SD using Hozo’s method [16]. The analysis was performed using Review Manager (RevMan) 5.4 software (The Cochrane Collaboration, Oxford, UK) [17].

Risk of Bias and Quality of Evidence Assessment

The risk of bias in the included articles was assessed using the “risk of bias in nonrandomized studies - of intervention” (ROBINS-I). This tool encompasses seven domains of potential biases at three different stages of the study: confounding and selection biases in the preintervention phase, classification bias during the intervention, deviations from the intended intervention, missing data, measurement of outcomes, and selection of reported result biases in the postintervention phase [18]. The quality of the evidence was assessed using the standards from the Grading of Recommendations Assessment, Development, and Evaluation (GRADE) for each outcome using the GRADEpro - Guideline Development Tool software (Evidence Prime, Hamilton, Canada, USA) [19].

Results

Study Identification and Selection

**Figure 1:**
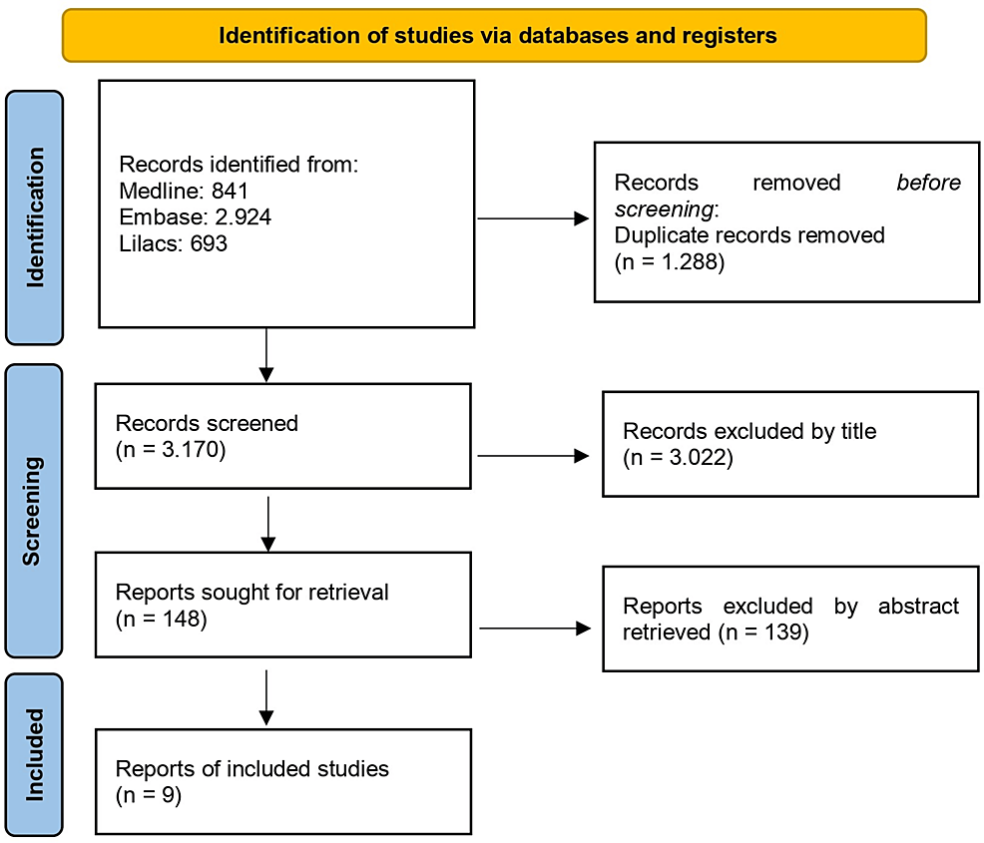
Study selection flowchart according to the PRISMA guidelines PRISMA: Preferred Reporting Items for Systematic Reviews and Meta-Analysis

The initial research yielded 4.458 articles. A total of 1.288 studies were excluded due to duplication, and 3.161 were excluded based on the title and abstract. This resulted in a total of nine articles (Figure 1).

Study Characteristics

Data from nine observational studies, comprising 897 patients, were processed for quantitative analysis (Table 1).

**Table 1:** Details of the included studies R0: complete resection; EA: endoscopic approach; SA: surgical approach; AE: adverse events; Major AE: Clavien-Dindo ≥ III; NA: data not available

Study	Region	Total	EA	SA	R0	AE	Major AE	Recurrence	Mortality	Hospital stay
Abe et al. 2022 [13]	Japan	74	43	31	EA: 21/43; SA: 31/31	NA	EA: 10/43; SA: 10/31	EA: 7/43; SA: 1/31	EA: 0/43; SA: 1/31	EA: 11 (7-57); SA: 42 (14-68)
Seyfried et al. 2022 [20]	Germany	85	42	43	EA: 41/42; SA: 43/43	EA: 40/42; SA 41/43	EA: 12/42; SA: 10/43	EA: 3/42; SA: 0/43	EA: 0/42; SA: 3/43	EA: 6.5 ± 7.6; SA: 20.2 ± 12
Haraldsson et al. 2021 [7]	Sweden	172	55	117	EA: 21/55; SA: 117/117	NA	NA	NA	EA: 0/55; SA: 0/117	NA
Dubois et al. 2016 [21]	Switzerland	30	11	19	EA: 5/11; SA: 1/19	EA: 1/11; SA: 13/19	EA: 0/11; SA: 6/19	NA	EA: 0/11; SA: 0/19	EA: 0; SA: 14 (10-30)
Onkendi et al. 2014 [22]	USA	180	130	50	EA: 121/130; SA: 50/50	EA: 38/139; SA: 29/50	NA	EA: 44/130; SA: 3/50	EA: 0/130; SA: 1/50	NA
Ceppa et al. 2013 [23]	USA	109	68	41	EA: 54/68; SA: 37/41	EA: 12/68; SA: 17/41	NA	NA	EA: 0/68; SA: 0/41	EA: 0.6 ± 268; SA: 10.1 ± 1
Kim et al. 2013 [24]	South Korea	91	57	34	EA: 44/57; SA: 33/34	NA	NA	EA: 7/57; SA: 0/34	EA: 0/57; SA: 0/34	NA
Irani et al. 2009 [25]	USA	123	102	21	EA: 88/102; SA: 21/21	NA	NA	EA: 8/102; SA: 0/21	EA: 0/102; SA: 0/21	NA
Kim et al. 2009 [26]	South Korea	33	20	13	EA: 12/20; SA: 13/13	EA: 1/20; SA: 1/13	NA	EA: 6/20; SA: 4/13	EA: 1/20; SA: 1/13	NA

Risk of Bias and Quality of Evidence

For a comprehensive assessment of the overall quality of each outcome analysis, we followed the GRADE standards [27]. We utilized GRADEpro software, a tool for developing guidelines (Table 2).

**Table 2:** Assessment of the strength of recommendation and quality of evidence using GRADE a: According to Risk of Bias-2 (Rob-2) b: 50% < I2 < 75% GRADE: Grading of Recommendations Assessment, Development, and Evaluation

Certainty assessment							No. of patients		Effect		Certainty
No. of studies	Study design	Risk of bias	Inconsistency	Indirectness	Imprecision	Other considerations	Endoscopy	Surgery	Relative (95% CI)	Absolute (95% CI)	
Complete Resection											
9	observational studies	serious^a^	serious^b^	not serious	not serious	none	358/537 (66.7%)	363/369 (98.4%)	RR 0.72	275 fewer per 1.000 (from 413 fewer to 108 fewer)	⨁⨁◯◯
									(0.58 to 0.89)		Low
Recurrence											
6	observational studies	serious^a^	not serious	not serious	serious^b^	strong association	75/392 (19.1%)	8/192	RR 3.32	97 more per 1.000 (from 14 more to 302 more)	⨁⨁⨁◯
								-4.20%	(1.33 to 8.25)		Moderate
Overall Adverse Events											
5	observational studies	serious^a^	very serious^b^	not serious	not serious	none	92/280 (32.9%)	101/166 (60.8%)	not estimable	220 more per 1.000	⨁◯◯◯
										(from 0 fewer to 430 more)	Very low
Major Adverse Events											
3	observational studies	serious^a^	not serious	not serious	serious^b^	strong association	22/96 (22.9%)	26/93 (28.0%)	not estimable	110 more per 1.000	⨁⨁⨁◯
										(from 100 fewer to 320 more)	Moderate
Mortality											
9	observational studies	serious^a^	not serious	not serious	serious^b^	none	1/537 (0.2%)	6/373 (1.6%)	not estimable	10 more per 1.000	⨁⨁◯◯
										(from 10 fewer to 20 more)	Low
Length of Hospital Stay											
4	observational studies	serious^a^	very serious^b^	not serious	not serious	none	164	138	-	MD 14.69 lower	⨁◯◯◯
										(19.91 lower to 9.47 lower)	Very low

**Figure 2:**
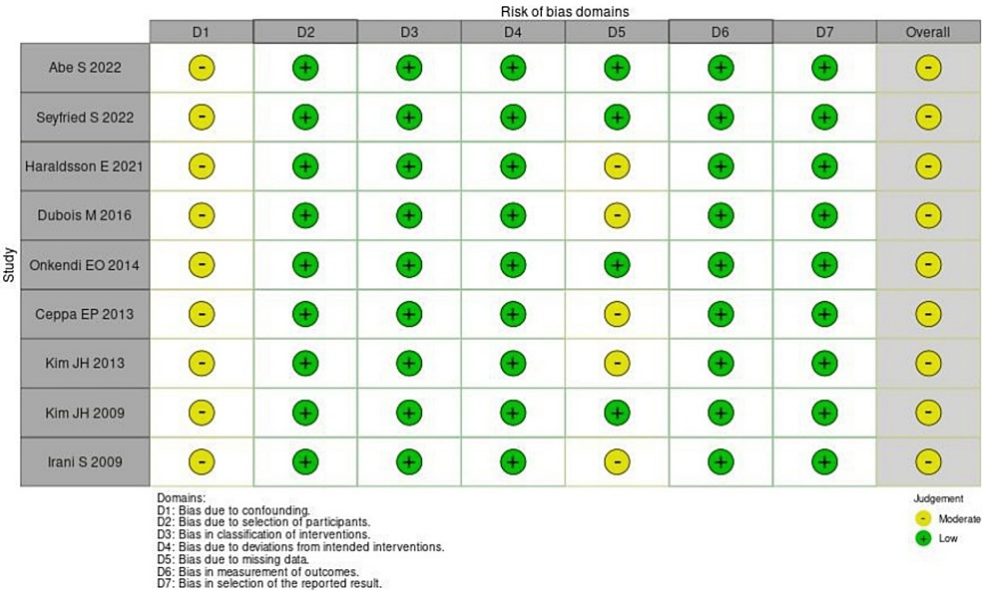
Risk of bias assessment using ROBINS-I [7,13,20-26] ROBINS-I: risk of bias in nonrandomized studies - of intervention

In the study, all the included studies had a moderate risk of bias by the ROBINS-I assessment (Figure 2).

Meta-analysis

Complete Resection

**Figure 3:**
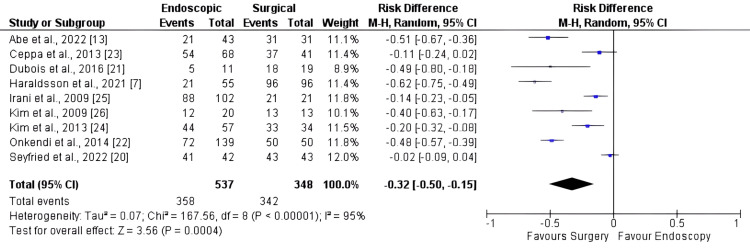
Forest plot for complete resection

Nine studies, comprising 897 patients, were included in the complete resection analysis. The surgical method exhibited a higher rate of primary resection than the EA (Figure 3), with an RD of -0.32 [95% CI: -0.50, -0.15; I2: 95%; p < 0.001].

Recurrence

**Figure 4:**
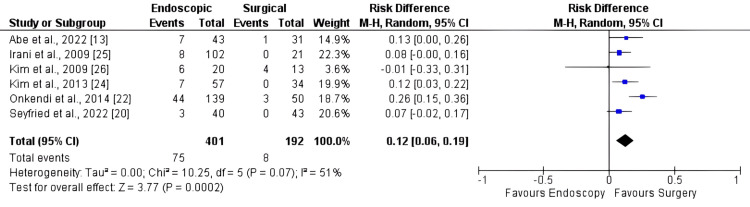
Forest plot for recurrence

Six studies, comprising 584 patients, were included in the recurrence analysis. There was a higher recurrence rate in endoscopy than in surgery (Figure 4), with an RD of 0.12 [95% CI: 0.06, 0.19; I2: 51%; p < 0.001].

Overall Adverse Events

**Figure 5:**
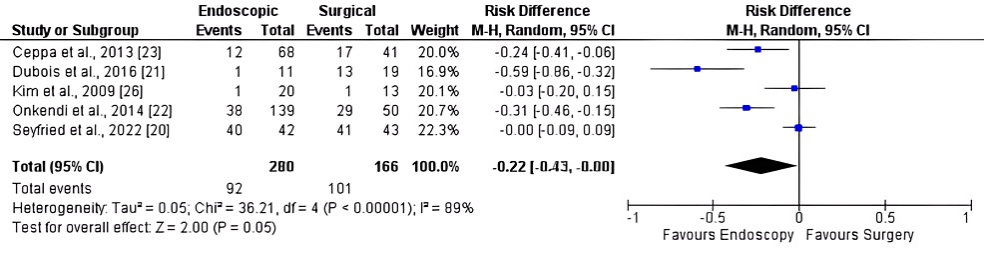
Forest plot for overall adverse events

Six studies, comprising 446 patients, were included in the adverse event analysis. There was a higher risk of adverse events in surgery compared to endoscopy (Figure 5), with an RD of -0.22 [95% CI: -0.43, 0.00; I2: 89%; p = 0.05].

Major Adverse Events

**Figure 6:**
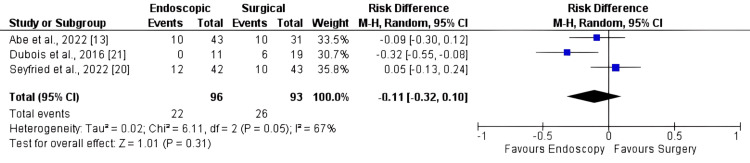
Forest plot for major adverse events

The evaluation of major complications was reported in three articles including 189 patients. No difference was noted between endoscopy and surgery (Figure 6), with an RD of -0.11 [95% CI: -0.32, 0.10; I2: 67%; p = 0.31].

Mortality

**Figure 7:**
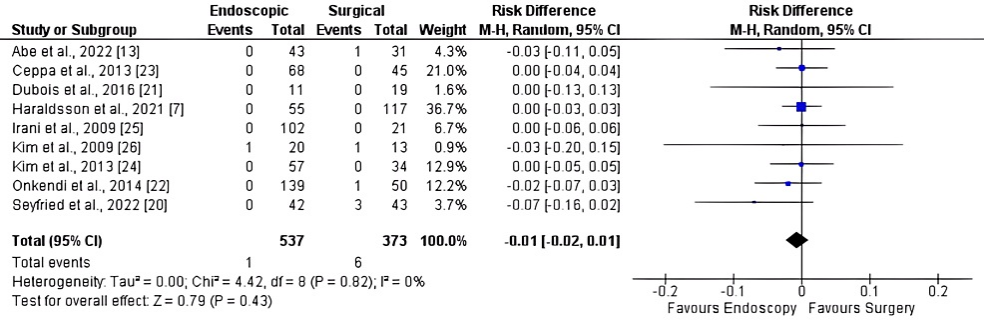
Forest plot for mortality

Mortality was reported in all articles, which comprised 910 patients. The RD was -0.01 [95% CI: -0.02, 0.01; I2 = 0%, p = 0.43]. No significant differences in mortality were found between the two approaches (Figure 7).

Length of Hospital Stay

**Figure 8:**
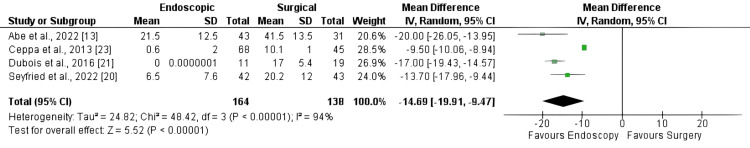
Forest plot for length of hospital stay

The length of hospital stay was found in four articles, which included 302 patients. The MD was -14.69 [95% CI: -19.91, -9.47; I2=94%, p < 0.001]. The results demonstrate a significantly shorter length of hospital stay with the EA (Figure 8).

Discussion

Duodenal papillary lesions constitute a rare entity but are increasingly diagnosed. Although predominantly benign, many ALs have malignant potential and can cause complications such as cholangitis or pancreatitis. Consequently, the treatment of ALs through EA, SA, or PD is recommended in most cases [21,24-26]. Randomized controlled trials (RCTs) comparing EA, SA, or PD and AL treatment for noninvasive lesions are lacking and may not be conducted in the future, because they are uncommon and have very variable presentations. The expertise and approach of surgeons and endoscopists are also very variable [28]. The current choice of treatment relies on expert opinion and the availability of endoscopic or surgical resources and is not guided only by high-quality evidence-based guidelines. This study represents a pioneering systematic review aimed at comparing SAs and EAs for duodenal papillary lesions.

A crucial step in determining AL management relies on the determination of indications for endoscopic intervention [22]. Although it is widely acknowledged that endoscopic papillectomy (EP) should be reserved for cases where the adenoma is localized to the ampullary region, the specific criteria guiding these indications remain unclear. The European Society of Gastrointestinal Endoscopy (ESGE) latest guideline published in 2021 considers surgical intervention for patients with a diverticulum, tumors larger than 4 cm, or intraductal involvement exceeding 20 mm [1]. However, these recommendations were accompanied by a low level of evidential support, thereby leading to a scenario where each medical facility customizes its approach based on individual experiences and resource availability. While the distinguishing criteria between endoscopic and surgical procedures remain somewhat uncertain [9], the analysis of the articles suggests that the SA may have higher complication risks. This is observed despite a higher resection rate, indicating that for smaller lesions, the EA may be the preferred choice.

Out of the nine articles analyzed, only six reported on studies in which follow-up was conducted, and all of the studies lacked a standardized protocol. Albeit with a low level of evidence, ESGE strongly recommends follow-up of these patients at three months, six months, and 12 months, followed by annual follow-ups until five years of monitoring is complete [1].

R0 resection rates are higher and recurrence rates are lower after surgical treatment. However, when histology proves to be benign, recurrence is ultimately treated by endoscopy, which is minimally invasive and effectively resolves the issue in the majority of instances [13,7,23].

As previously acknowledged, surgical procedures are known to carry a higher risk of complications [2,3,29,30]. However, our findings indicate no significant difference in the overall rates of adverse events or major adverse events. The results regarding mortality suggest that both SAs and EAs for duodenal papillary lesions appear to be viable, as there were no significant differences observed between the two strategies. It is noteworthy that the p-value was 0.05, indicating a trend toward more adverse events with surgery. This aligns with existing literature that reinforces such a tendency.

Furthermore, our study has demonstrated a shorter hospital stay after endoscopic treatment [13,20,21,23]. The prospect of reducing the length of hospital stays and post-treatment complications associated with the EA may lead to substantial and sustainable long-term cost savings. However, it is important to note that the available literature does not address this aspect, precluding definitive statements on the matter.

Our study has several limitations, beginning with the rarity of duodenal papillary lesions and the variety of presentations [20,31]. However, this aligns with what we have observed in clinical practice, given the multiple presentations of tumors and their distinct characteristics. The predominant source of data in our analysis derives from single-center retrospective cohort studies, given the notable scarcity of comparative RCTs - a limitation arising from the inherent challenges in conducting randomized studies on this subject. As previously exposed in the discussion, the studies included in our analysis exhibit a wide array of designs and qualities owing to the low prevalence and the diverse clinical presentations of the pathology, making randomization practically unfeasible. This is further compounded by the substantial variation in experience among endoscopy teams at each center. Another noteworthy aspect is the lack of standardization in the adverse effects reported in the articles; only in three studies was it possible to establish a consistent pattern of complications.

To achieve a higher level of evidence, an alternative approach would involve homogeneous indications controlled in large centers (multicenter study) with prospective and relatively standardized evaluations. We acknowledge the limitations arising from the heterogeneity of the studied lesions and the absence of randomization. Given these circumstances, conducting an RCT may prove impractical. However, to address these limitations and enhance the robustness of the results, we suggest considering a study design that involves more homogeneous indications, conducted in large centers, with prospective evaluations and more standardized protocols. This alternative approach can contribute to a higher degree of confidence in the results, despite the inherent limitations in the field of study.

## Conclusions

As expected, our data suggest a higher complete resection rate and lower recurrence from surgical interventions, but this is associated with an elevated risk of adverse events and a longer hospital stay. Consequently, for smaller and benign lesions or in patients who are not suitable candidates for surgery, endoscopy can represent a safe and effective alternative. However, it's imperative for clinicians to carefully weigh the benefits and risks of each approach, considering factors such as lesion size, location, and patient comorbidities, to determine the most appropriate course of action for optimal patient outcomes.

## References

[1] Vanbiervliet G, Strijker M, Arvanitakis M (2021). Endoscopic management of ampullary tumors: European Society of Gastrointestinal Endoscopy (ESGE) Guideline. Endoscopy.

[2] Mendonça EQ, Bernardo WM, Moura EG, Chaves DM, Kondo A, Pu LZ, Baracat FI (2016). Endoscopic versus surgical treatment of ampullary adenomas: a systematic review and meta-analysis. Clinics (Sao Paulo).

[3] Heise C, Abou Ali E, Hasenclever D (2020). Systematic review with meta-analysis: endoscopic and surgical resection for ampullary lesions. J Clin Med.

[4] Bohnacker S, Seitz U, Nguyen D (2005). Endoscopic resection of benign tumors of the duodenal papilla without and with intraductal growth. Gastrointest Endosc.

[5] Espinel J, Pinedo E, Ojeda V, Guerra Del Río M (2016). Endoscopic ampullectomy: a technical review. Rev Esp Enferm Dig.

[6] Schneider L, Contin P, Fritz S, Strobel O, Büchler MW, Hackert T (2016). Surgical ampullectomy: an underestimated operation in the era of endoscopy. HPB (Oxford).

[7] Haraldsson E, Halimi A, Rangelova E, Valente R, Löhr JM, Arnelo U (2022). Adenomatous neoplasia in the papilla of Vater endoscopic and/or surgical resection?. Surg Endosc.

[8] Nappo G, Gentile D, Galvanin J (2020). Trans-duodenal ampullectomy for ampullary neoplasms: early and long-term outcomes in 36 consecutive patients. Surg Endosc.

[9] Sakai A, Tsujimae M, Masuda A (2019). Clinical outcomes of ampullary neoplasms in resected margin positive or uncertain cases after endoscopic papillectomy. World J Gastroenterol.

[10] Taliente F, Bianco G, Moschetta G, Franco A, Giovinazzo F, Agnes S, Spoletini G (2022). From endoscopic resection to pancreatoduodenectomy: a narrative review of treatment modalities for the tumors of the ampulla of Vater. Chin Clin Oncol.

[11] De Palma GD (2014). Endoscopic papillectomy: indications, techniques, and results. World J Gastroenterol.

[12] Vu Trung K, Heise C, Abou-Ali E (2024). Endoscopic papillectomy for ampullary lesions of minor papilla. Gastrointest Endosc.

[13] Abe S, Sakai A, Masuda A (2022). Advantage of endoscopic papillectomy for ampullary tumors as an alternative treatment for pancreatoduodenectomy. Sci Rep.

[14] Liberati A, Altman DG, Tetzlaff J (2009). The PRISMA statement for reporting systematic reviews and meta-analyses of studies that evaluate healthcare interventions: explanation and elaboration. BMJ.

[15] Clavien PA, Barkun J, de Oliveira ML (2009). The Clavien-Dindo classification of surgical complications: five-year experience. Ann Surg.

[16] Hozo SP, Djulbegovic B, Hozo I (2005). Estimating the mean and variance from the median, range, and the size of a sample. BMC Med Res Methodol.

[17] (2020). Review Manager (RevMan). Collaboration.

[18] Sterne JA, Hernán MA, Reeves BC (2016). ROBINS-I: a tool for assessing risk of bias in non-randomised studies of interventions. BMJ.

[19] Bezerra CT, Grande AJ, Galvão VK, Santos DH, Atallah ÁN, Silva V (2022). Assessment of the strength of recommendation and quality of evidence: GRADE checklist. A descriptive study. Sao Paulo Med J.

[20] Seyfried S, Kähler G, Belle S (2022). Endoscopic papillectomy or pancreaticoduodenectomy for ampullary lesions: a single center retrospective cohort study. Scand J Gastroenterol.

[21] Dubois M, Labgaa I, Dorta G (2017). Endoscopic and surgical ampullectomy for non-invasive ampullary tumors: short-term outcomes. Biosci Trends.

[22] Onkendi EO, Naik ND, Rosedahl JK (2014). Adenomas of the ampulla of Vater: a comparison of outcomes of operative and endoscopic resections. J Gastrointest Surg.

[23] Ceppa EP, Burbridge RA, Rialon KL (2013). Endoscopic versus surgical ampullectomy: an algorithm to treat disease of the ampulla of Vater. Ann Surg.

[24] Kim HN, Kim KM, Shin JU (2013). Prediction of carcinoma after resection in subjects with ampullary adenomas on endoscopic biopsy. J Clin Gastroenterol.

[25] Irani S, Arai A, Ayub K (2009). Papillectomy for ampullary neoplasm: results of a single referral center over a 10-year period. Gastrointest Endosc.

[26] Kim JH, Kim JH, Han JH, Yoo BM, Kim MW, Kim WH (2009). Is endoscopic papillectomy safe for ampullary adenomas with high-grade dysplasia?. Ann Surg Oncol.

[27] Guyatt GH, Oxman AD, Schünemann HJ, Tugwell P, Knottnerus A (2011). GRADE guidelines: a new series of articles in the Journal of Clinical Epidemiology. J Clin Epidemiol.

[28] Scroggie DL, Mavroeidis VK (2021). Surgical ampullectomy: a comprehensive review. World J Gastrointest Surg.

[29] Klein A, Qi Z, Bahin FF (2018). Outcomes after endoscopic resection of large laterally spreading lesions of the papilla and conventional ampullary adenomas are equivalent. Endoscopy.

[30] El Hajj II, Coté GA (2013). Endoscopic diagnosis and management of ampullary lesions. Gastrointest Endosc Clin N Am.

[31] Hollenbach M, Ali EA, Auriemma F, Gulla A, Heise C, Regnér S, Gaujoux S (2020). Study protocol of the ESAP study: endoscopic papillectomy vs. surgical ampullectomy vs. pancreaticoduodenectomy for ampullary neoplasm-a pancreas2000/EPC study. Front Med (Lausanne).

